# Medico-Geological Studies on Pulmonary Phthisis and Typhoid Fever, in Their Relations to Marshy Districts

**Published:** 1846-07

**Authors:** 


### Art. XIX.

***Etudes de Géologie Médicale sur la Phthisie Pulmonaire at la Fièvre Typhoide, dans leurs Rapports avec les Localités Marécageuses, &c.*** Par S. Ch. Boudin, &c.—*Paris,* 1845. 8vo, pp. 79.

***Medico-Geological Studies on Pulmonary Phthisis and Typhoid Fever, in their Relations to Marshy Districts.*** By S. Ch. Boudin, &c.—*Paris,* 1845. 8vo, pp 79.

The author of this pamphlet has had opportunities, in the military service of his country, to obtain personal information on the subject which he has discussed. He has served in Greece; he has been surgeon to various military hospitals in Algeria, and he is now surgeon-general to the military hospital at Versailles. It is not surprising that, with these opportunities, this essay should have obtained the prize for the best essay on public hygiene, given by the editors of the 'Annales d'Hygiène Publique et Médecine Légale,' in the year 1844.

It was in 1828, while serving with the French expedition to the Morea, that M. Boudin first noticed the remarkable antagonism between typhoid and pulmonary affections on the one hand, and marsh fevers on the other.

In 1832 he was appointed medical director of the quarantine hospital at Marseilles, and he had again occasion to notice that the troops debarked from the marshy localities of Greece and Algeria were rarely affected with either typhus or phthisis—the two diseases to which a great proportion of the mortality amongst French troops is attributed, and which "frightfully decimated" the garrison at Marseilles. In 1841 M. Boudin published his 'Traité des Fièvres Intermittentes,' in which he discussed this antagonism; and again in 1843, at greater length, in his 'Essai de Géographie Médicale.' A few days after the latter appeared, M. Rayer proposed to the Royal Academy of Medicine of Paris that a physician lately gone to Algeria should be instructed to inquire into the relations of phthisis to the marshy localities of that country. The proposition was unanimously adopted, and the subject became at once popular—nay, according to our author, just as every political question agitated in the French parliament finds an echo throughout the whole world, so the question how far marshy localities exercise a remedial influence was re-echoed beyond the Alps, at Vienna, at Berlin, and on the borders of the Thames, so soon as the French took it up.

In the introduction to his essay, M. Boudin, in making these statements, adds the names of several French authors on the subject, and mentions several facts which had come under his own observation during his services in the army. In the second chapter he discusses the question of antagonism in general, which he defines as the principle in virtue of which a diathesis or morbid state affords a protection more or less complete against the manifestation of certain morbid conditions. Examples of such antagonism are presented in the action of specifics, as quinine and arsenic, in the treatment of ague; or of prophylactics, as belladonna, in the prevention of scarlatina [which prophylaxis we entirely doubt]. Concurrently with this protection from certain morbid states, there may be a predisposition to others. Geological and climacteric influences are important agents in this respect, by determining the nature and kind of food, dwelling, clothing, employments, and other agents affecting the hygienic condition. M. Boudin observes that a glance at a geological map of England will suffice to show that the largest and most populous towns of England are situate on the hard red sandstone; the agricultural and pastoral towns on the chalks or oolites.

The influence of a previous residence is felt after removal from a district having certain hygienic relations. The English sweating sickness is quoted as an example of this general proposition. M. Boudin relates several more modern examples of the same kind. Towards the end of April, 1843, two infantry regiments arrived at Courbevoie (Versailles), the one (the 23d) from the north of France, the other (the 69th) from the citadel of Strasburg, the focus of a marshy district, where it had been quartered for two years. These two regiments occupied the same barracks, and were in every respect situate in the same circumstances. Yet the disease that affected each regiment differed widely in character. The 23d sent to the hospital cases of pulmonary inflammations and typhoid fevers; the 69th filled it for more than a year with intermittents, the disease attacking even those who remained free from it all the time they were at Strasburg. Another regiment, which subsequently arrived at Courbevoie from Strasburg, presented cases of disease identical with the preceding.

This predisposition is not confined to men. For several years the mean annual loss of horses by disease in the French army amounted to 197 per 1000, In the Prussian service it is only 20 per 1000; and in the French Gendarmerie is only 14 per 1000. This led M. Boudin to inquiries, and he soon found a case in point. Two cavalry regiments, towards the end of 1841, arrived from different quarters at Versailles, where they were placed under circumstances in all respects alike. Their sanitary condition was, however, very different. From the 1st October, 1841, to 1st December, 1843, the deaths of the horses in the two regiments were as follows:

| | Number of horses sick. | Number of horses killed or died. | Numbers killed with glanders. | Numbers killed with farcy. |
|---|---|---|---|---|
| Cuirassiers . | 513 | 180 | 93 | 28 |
| Hussars . | 214 | 48 | 12 | 3 |
| | — | — | — | — |
| Difference . | 299 | 132 | 81 | 25 |

That this enormous difference in the sanitary condition of the horses in the two regiments was not owing to the difference of service, is proved by the fact that in the whole army the cuirassiers lost 18 per cent. of their horses, and the hussars 16½. M. Boudin thinks it can only be attributed to the circumstances in which the regiments were respectively placed *before* their arrival at Versailles. The hussars had been in the more comfortable garrisons of the east of France; the cuirassiers had been quartered in those of the north, which are considered inferior to the former, especially in the quality of the forage.

In the third chapter M. Boudin inquires more particularly into our Dr. Wells's doctrine of antagonism between intermittents and phthisis, and the facts which elucidate it. A river during a long course will present examples of this kind. Where it flows rapidly and within its banks, we should expect pulmonary phthisis and typhoid fevers to prevail; but at those points in which it spreads out, in a flat country, intermittents will supersede the latter. The Rhine, in its long course, presents an instance of this kind. Numerous other illustrations are brought forward by M. Boudin, particularly from our army statistical reports, to which we have previously called the attention of our readers. From these and other facts it is very clearly shown, that at our various stations in India, North America, and Africa, where intermittents are prevalent, pulmonary diseases are rare, and *vice versâ*. The statistics of the United States' army indicate the same antagonism. In Algeria, intermittents and other forms of marsh fever are extremely common. From April to October, 1308 cases of fever were admitted into the military hospital at Algiers; of these, 288 were intermittents, (quotidian?) 239 tertians, 92 remittents, and 106 pseudo-continued. The hospitals at Medeah, Blidah, and Bona present the same proportions nearly. When the resumption of hostilities by Abd-el-Kader rendered it necessary to send reinforcements to Africa, typhoid cases became more frequent, and amounted to 1 in 28 of the total admissions; but it was remarkable that no soldier who had been more than eight months in Africa was attacked with typhus.

M. Boudin passes from Africa to the marshy grounds of Europe, and especially to those of his own country; and brings together a multitude of facts, all tending to the establishment of the same principle, namely, that there is a real antagonism between the two classes of diseases, and that a residence in a marshy district is, within certain limits, prophylactic or curative of phthisis pulmonalis, or protects against typhoid fevers. Remarkable examples are given of the latter part of the proposition. One or two of these we shall note; they are from the 'Géographie Médicale' of our author. In August, 1841, the garrison at Marseilles suffered so severely from typhus, that five of every seven admitted into hospital were affected by it. A regiment arrived from Africa at this time, and was quartered for twelve days at Marseilles. During that period it sent 49 fever cases to the hospital, but they were all intermittents of various types. In the beginning of the year 1842, another regiment came to Marseilles from Africa, and was in garrison there for about five months. Not one case of typhus occurred in it, although other battalions suffered as usual. Individual examples of the cure of phthisis, after residence in a marshy district, are given by M. Boudin, as communicated to him by those who observed them.

M. Boudin then proceeds to demonstrate the antagonism of races, pointing out, in particular, the greater liability of the white troops in the West Indies to yellow fever, of the black troops to pulmonary diseases, and *vice versâ*. He then notices the antagonism of the sexes, and of different periods in history and of the year. Under these heads free use is made of British national statistics, both civil and military. The "conclusions" we give in M. Boudin's own words:

"1. Those localities in which the producing cause of endemic intermittents thoroughly modify the constitution of man are remarkable for the infrequency of pulmonary phthisis and typhoid fever.

"2. The localities in which pulmonary phthisis and typhoid fever are particularly prevalent are remarkable for the infrequency and mildness of intermittent fevers *contracted on the spot.*

"3. The drying up of a marsh, or its conversion into a lake, diminishes or prevents intermittent fevers, but seems to dispose the organism to a new series of diseases, in which pulmonary phthisis and (according to the climate) typhoid fever are particularly prominent.

"4. After a residence in a thoroughly marshy locality, an individual enjoys an immunity from typhoid fever, the degree and duration of which is in direct proportion, 1st, to the length of the previous residence; 2d, to the intensity of the fevers proper to the locality, considered under the twofold relations of form and type; 3d, or, in other words, that a residence in a country of remittent and continued fevers, such as certain points of the coast of Algeria, and the centre of the marshy part of Brasse, is more prophylactic against the disease referred to than, for example, a residence near the marshy embouchure of the Bièvre at Paris.

"5. The conditions of latitude and longitude, and of height [above the sea], which limit the manifestation of marsh fevers, equally limit the curative or prophylactic influence of the marsh miasm.

"6. Lastly, certain conditions of race, and possibly of sex, diminish the susceptibility of the system to the cause of marsh fevers, and in an equal degree diminish the therapeutic influence of that cause." (p. 76.)

The subject of which M. Boudin treats has considerable practical value in the distribution of troops, and in the hygiene of those predisposed to consumption. Although we hope this "echo from the banks of the Thames" may gratify our author, we must observe that it is an old subject of discussion in England, and the practical value of our empirical knowledge has in some degree been tested. We would recommend more of our

English authors to M. Boudin for perusal—especially Sir James Clark's work on Climate. He will there find that his theoretical views are not borne out altogether by facts. We do not say this testily, but with a kindly feeling, and a wish that M. Boudin will continue to work at his problem, for we are sure there is something in it, although not that which he anticipates. We apply this remark as well to the supposed antagonism of marsh and typhoid fevers, as of marsh fevers and phthisis.

---

